# Resolution of metastatic cutaneous Crohn's disease with upadacitinib monotherapy

**DOI:** 10.1016/j.jdcr.2024.02.017

**Published:** 2024-03-04

**Authors:** Kevin M. Burningham, Kritin K. Verma, Anisha B. Patel, Stephen K. Tyring

**Affiliations:** a Center for Clinical Studies, Ltd., Webster, Texas; b Texas Tech University Health Sciences Center School of Medicine, Lubbock, Texas; c Department of Dermatology, the University of Texas Health Science Center at Houston, Bellaire, Texas; d Department of Dermatology, the University of Texas MD Anderson Cancer Center, Houston, Texas

**Keywords:** case report, inflammatory bowel disease, Janus kinase-1 inhibitor, metastatic cutaneous Crohn's disease, upadacitinib

## Introduction

Metastatic Crohn’s disease (MCD) is the rare appearance of skin lesions with histopathologic features of Crohn’s disease (CD) but separated from the gastrointestinal tract by normal organ systems.^1^ It causes significant morbidity and has no standardized treatment strategy due to the paucity of cases.^1^ Here, we present a case of MCD that was successfully treated with upadacitinib monotherapy, an orally bioavailable selective Janus kinase-1 (JAK-1) inhibitor.

## Case report

**Fig 1:**
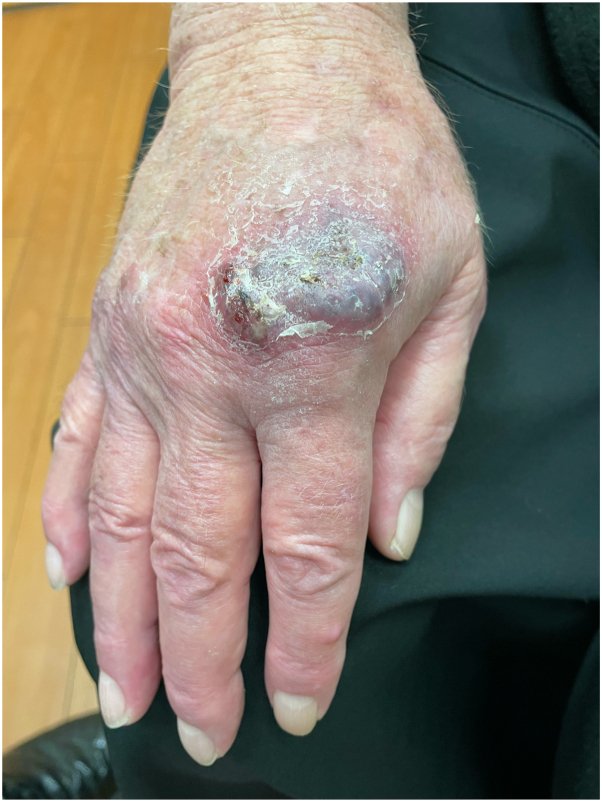
Metastatic cutaneous Crohn’s disease. Scaly, violaceous, indurated, painful, and pruritic plaque with erosions and erythema overlying the second metacarpal–phalangeal joint of the right hand.

A 63-year-old White man with a 19-year history of CD presented to the dermatology clinic with an 8-month history of worsening red “pimples” progressing into painful purple plaques affecting both forearms and hands. A physical examination revealed normal vitals and multiple erythematous pink–red papules without significant epidermal involvement and tender, violaceous indurated plaques with an overlying scale and surrounding erythema (Fig 1).

The patient denied systemic symptoms, recent illness, insect bites, trauma to the affected areas, and any changes in exposures. He denied a history of other skin disorders. His CD had proven refractory to multiple treatment modalities, including methotrexate, rifaximin, infliximab, and adalimumab. His disease course was previously complicated by multiple entero- and recto-cutaneous fistulae status postexploratory laparotomy, colostomy diversion, and multiple fistulectomies. At time of presentation, the patient’s gastrointestinal CD symptoms were moderate but stable over the previous year, and he had discontinued all CD therapy.

**Fig 2:**
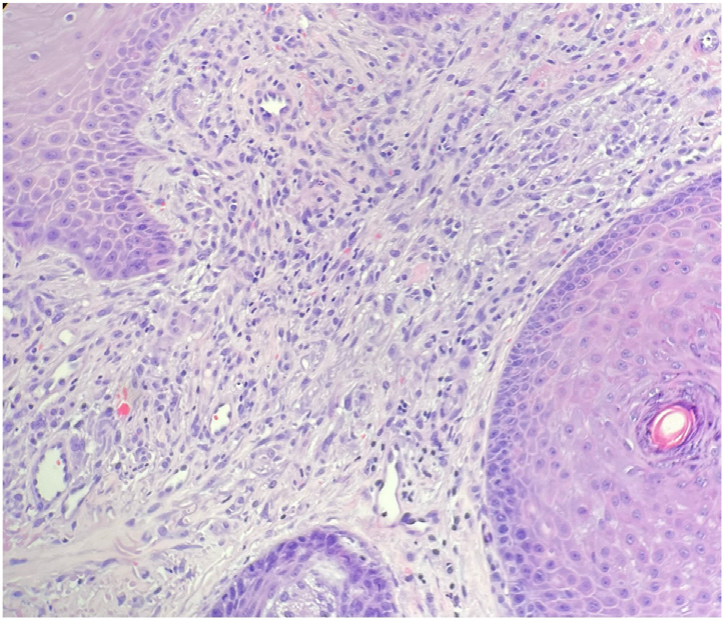
Metastatic cutaneous Crohn’s disease. Granulomatous dermatitis with multinucleated histiocytes. (Hematoxylin-eosin stain; original magnifications: ×200.)

**Fig 3:**
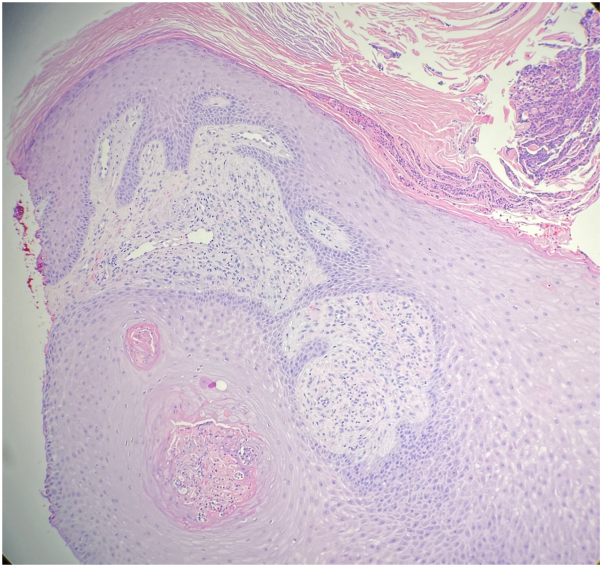
Metastatic cutaneous Crohn’s disease. Suppurative dermatitis with pseudoepitheliomatous hyperplasia. (Hematoxylin-eosin stain; original magnifications: ×100.)

Two 4-mm punch biopsies were performed of representative lesions. Histologic evaluation revealed pseudoepitheliomatous hyperplasia with sinus tracts and suppurative granulomatous dermatitis. The first biopsy demonstrated predominantly multinucleated histiocytes without evidence of sinus tract rupture and only minimal neutrophilic infiltrate (Fig 2). The second biopsy had more diffuse neutrophilic infiltrate, including subcorneal pustules and dermal suppuration, but still with the background of granulomatous dermatitis (Fig 3). Periodic acid-Schiff and Fite special stains were performed for both specimens and failed to reveal organisms.

The histologic differential diagnosis includes infection, reactive neutrophilic dermatosis, and metastatic cutaneous CD. Clinicopathologic correlation, considering the negative stains for organisms, favored a diagnosis of MCD.

**Fig 4:**
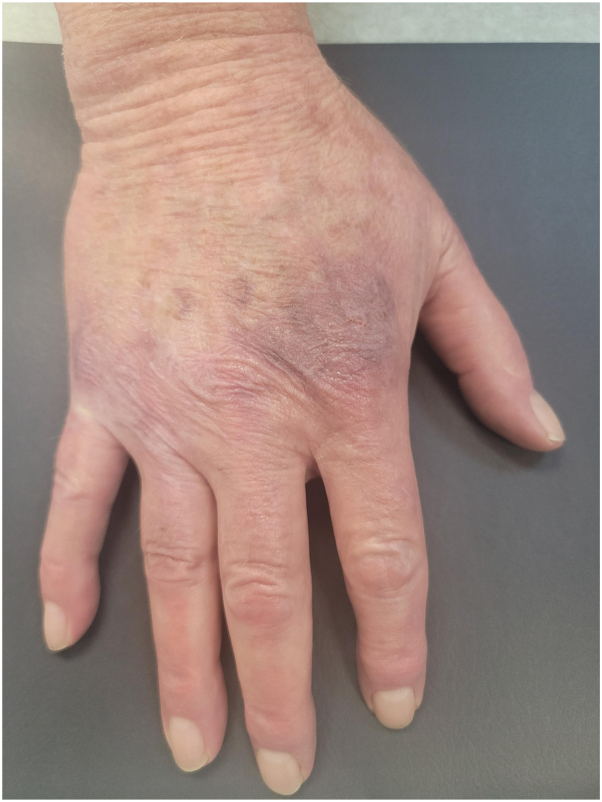
Metastatic cutaneous Crohn’s disease. The patient’s right hand following 4 months of upadacitinib monotherapy.

Considering the patient’s long history of CD refractory to adalimumab, we prescribed oral upadacitinib (30 mg once daily) as monotherapy for 4 weeks. The patient reported significant relief from gastrointestinal CD symptoms less than a week after beginning this treatment. At 2 weeks, he also reported improvement of his cutaneous lesions. After 4 weeks, the patient’s dose was increased to 45 mg once daily, the dosage indicated for gastrointestinal CD. The patient returned for a repeat evaluation 2 weeks after the dose increase, reporting dramatically decreased gastrointestinal CD symptoms. An examination revealed significant improvement of the cutaneous lesions, which were no longer indurated, scaly, tender, or pruritic. At 4 months after initiation of upadacitinib, the cutaneous lesions had completely cleared with residual postinflammatory hyperpigmentation (Fig 4). To date, the patient has reported no adverse effects of upadacitinib therapy, including infection or venous thromboembolism.

## Discussion

Between 22% and 44% of patients with CD experience cutaneous symptoms.^2^^,^^3^ Cutaneous CD usually occurs as a complication of the direct progression of gastrointestinal disease to the skin surface, causing ulcers or fistulae in the perioral, perianal, or abdominal regions.^4^ Meanwhile, MCD is an extremely rare extraintestinal manifestation of CD, with <100 cases reported.^5^ It is characterized by erythematous, painful skin lesions with histopathologic features of CD that are separated from the gastrointestinal tract by normal organ systems.^2^^,^^3^ Its morphology is highly variable in different patients and throughout the disease course, mimicking multiple other CD-related dermatoses,^3^ making histologic evaluation and exclusion of infection crucial to accurate diagnosis. In this case with neutrophilic infiltrate, infection and reactive neutrophilic dermatosis were considered less likely due to the negative organism stains and the predominance of multinucleated histiocytes in the first biopsy, respectively. Although some lesions have reportedly resolved spontaneously, they often persist and cause significant morbidity.^3^ MCD occurs in all age groups and equally in male and female patients.^5^ It appears to favor moist environments, such as the genitalia and flexural areas, although the extremities can be affected, as in our patient.^2^^,^^3^ The exact etiology of MCD is still being elucidated. Due to its low incidence, to our knowledge, no randomized controlled trials have been performed to date examining this disorder, and no standardized treatment strategy has been established.^5^ Several therapeutic approaches have been anecdotally reported with varying degrees of success, including oral and topical steroids, potassium permanganate, topical and systemic sulfasalazine, oral psoralen with UV-A, oral metronidazole, debridement/curettage, systemic immunomodulators cyclosporin, azathioprine, and 6-mercaptopurine, topical tacrolimus, infliximab, and adalimumab.^3^^,^^6^

Upadacitinib is an orally bioavailable selective JAK-1 inhibitor that disrupts inflammatory signals in the Janus kinase signal transducer and activator of transcription proteins (JAK-STAT) pathway.^7^ It was recently approved by the Food and Drug Administration for moderate–severe CD with inadequate response or intolerance to anti-tumor necrosis factor (TNF) agents, such as adalimumab.^7^ It has already been approved for multiple inflammatory conditions, including psoriatic arthritis, rheumatoid arthritis, atopic dermatitis, and ulcerative colitis.^8^ Upadacitinib's efficacy and safety have been studied in patients with an inadequate response to conventional synthetic disease-modifying antirheumatic drugs, such as methotrexate, biologic disease-modifying antirheumatic drugs, or at least one anti-TNF agent.^9^ Its promising results in these studies suggest that it could be an effective potential treatment for MCD.^9^ Agarwal et al^10^ recently reported mild improvement in a case of metastatic cutaneous CD treated with upadacitinib, in a patient concurrently receiving injections of ustekinumab, an anti–interleukin 12/interleukin 23 monoclonal antibody that is also indicated in moderate-to-severe CD refractory to anti-TNF agents. The authors believe that the present case is the first to report near-total resolution of MCD with upadacitinib therapy alone.

This 63-year-old patient with refractory MCD responded favorably to upadacitinib monotherapy, with significant improvement of cutaneous lesions after <2 months of treatment, complete resolution within 4 months, and no adverse effects to date. We suggest that upadacitinib could be a valuable option for clinicians treating MCD. Prospective studies to evaluate this possibility are warranted. It is crucial to promptly perform biopsy of suspicious cutaneous lesions in patients with known or suspected CD, to ensure timely diagnosis and early therapeutic intervention.

## Conflicts of interest

Dr Tyring is compensated by AbbVie, Inc. for professional presentations. Drs Burningham, Patel, and Mr Verma have no conflicts of interest to declare.
